# Prednisolone and acupuncture in Bell's palsy: study protocol for a randomized, controlled trial

**DOI:** 10.1186/1745-6215-12-158

**Published:** 2011-06-21

**Authors:** Feng Xia, Junliang Han, Xuedong Liu, Jingcun Wang, Zhao Jiang, Kangjun Wang, Songdi Wu, Gang Zhao

**Affiliations:** 1 Department of Neurology, Xijing Hospital, The Fourth Military Medical University, Xi'an, Shaanxi, China; 2 Department of Neurology, Central Hospital, Hanzhong, Shaanxi, China; 3 Department of Neurology, The First Hospital, Xi'an, Shaanxi, China

## Abstract

**Background:**

There are a variety of treatment options for Bell's palsy. Evidence from randomized controlled trials indicates corticosteroids can be used as a proven therapy for Bell's palsy. Acupuncture is one of the most commonly used methods to treat Bell's palsy in China. Recent studies suggest that staging treatment is more suitable for Bell's palsy, according to different path-stages of this disease. The aim of this study is to compare the effects of prednisolone and staging acupuncture in the recovery of the affected facial nerve, and to verify whether prednisolone in combination with staging acupuncture is more effective than prednisolone alone for Bell's palsy in a large number of patients.

**Methods/Design:**

In this article, we report the design and protocol of a large sample multi-center randomized controlled trial to treat Bell's palsy with prednisolone and/or acupuncture. In total, 1200 patients aged 18 to 75 years within 72 h of onset of acute, unilateral, peripheral facial palsy will be assessed. There are six treatment groups, with four treated according to different path-stages and two not. These patients are randomly assigned to be in one of the following six treatment groups, i.e. 1) placebo prednisolone group, 2) prednisolone group, 3) placebo prednisolone plus acute stage acupuncture group, 4) prednisolone plus acute stage acupuncture group, 5) placebo prednisolone plus resting stage acupuncture group, 6) prednisolone plus resting stage acupuncture group. The primary outcome is the time to complete recovery of facial function, assessed by Sunnybrook system and House-Brackmann scale. The secondary outcomes include the incidence of ipsilateral pain in the early stage of palsy (and the duration of this pain), the proportion of patients with severe pain, the occurrence of synkinesis, facial spasm or contracture, and the severity of residual facial symptoms during the study period.

**Discussion:**

The result of this trial will assess the efficacy of using prednisolone and staging acupuncture to treat Bell's palsy, and to determine a best combination therapy with prednisolone and acupuncture for treating Bell's palsy.

**Trial Registration:**

ClinicalTrials.gov: NCT01201642

## Background

Bell's palsy is an acute peripheral unilateral facial weakness or paralysis with an as yet unknown cause. Bell's palsy accounts for almost three quarters of peripheral facial palsies and the annual incidence is about 30 patients per 100 000. 71% of untreated patients with Bell's palsy will completely recover and 84% will have complete or near normal recovery. The remainder will have persistent moderate to severe weakness, facial contracture, or synkinesis [1-3]. Because of its unclear etiology, there has been longstanding controversy about what treatment should be given, with potential alternatives including corticosteroids, antiviral drugs, acupuncture and physiotherapy. Inflammation of the facial nerve is the most probable cause of Bell's palsy. Therefore corticosteroids are commonly used as treatment of Bell's palsy. A recent study shows significant short-term and long-term positive treatment effects of prednisolone in patients with Bell's palsy [4].

Acupuncture is a safe therapy with a low risk of adverse events in clinical practice [5]. It is one of the most commonly used treatments for Bell's palsy in China. Chinese traditional acupuncture is low cost and very convenient to local people. Though only limited experience has been reported with acupuncture for Bell's palsy [6], several studies provide increasing evidence for a beneficial effect of acupuncture as an adjunctive treatment of Bell's palsy [7-10]. Bell's palsy has different path-stages; it includes acute stage (1-7 days onset of disease), resting stage (8-20 days onset of disease) and restoration stage (21-90 days onset of disease) [11-14]. However, convincing evidence for the efficacy of staging acupuncture to treat Bell's palsy is lacking because of previous poor study designs, small sample sizes or unclear reporting treatment. It's not clear whether acupuncture is effective in different path-stages.

Thus, in view of these data, we designed this randomized controlled multicentre study of Bell's palsy patients, with the aim of comparing the effects of prednisolone and staging acupuncture in the recovery of the affected facial nerve. We also hoped to verify whether staging acupuncture along with prednisolone is more effective than prednisolone alone in larger number of patients, especially in terms of reducing their level of incapacity and improving their quality of life.

## Methods/Design

### Overview

**Figure 1:**
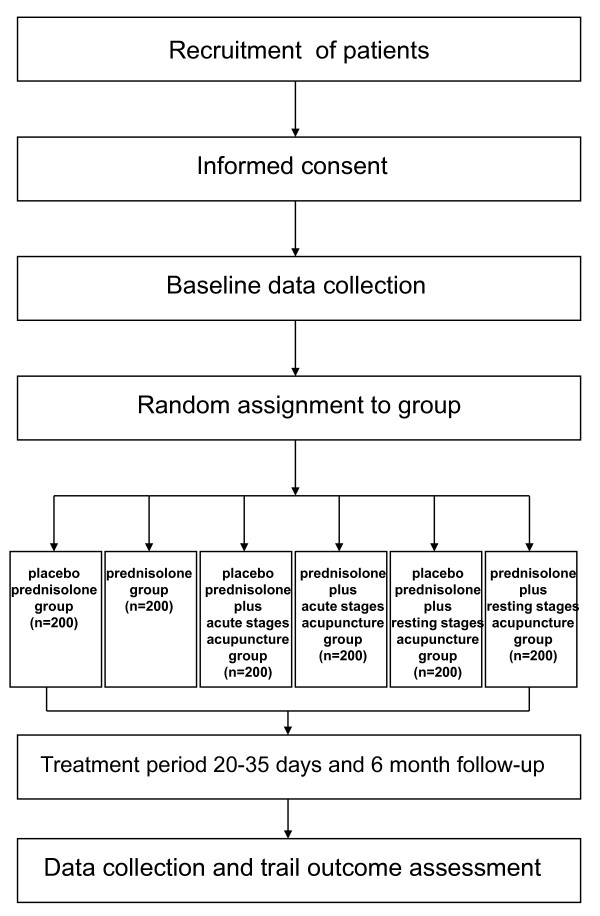
**Flow chart of the trial, describing the selection, randomization and follow-up process**.

This is a multicentre, randomized, placebo-controlled study (Figure 1). This study has been approved by the ethical committees of Xijing hospital and is registered with ClinicalTrials.gov database (reference no. NCT01201642). The study will be conducted according to the Declaration of Helsinki.

### Patients and enrolment

A power analysis is undertaken to calculate before conducting this trial: α = 0.05 and power = 80%. According to the previous literature, 70% of patients recover completely without treatment (placebo) compared with 80% of patients treated with prednisolone, acupuncture, or both. This difference of 10% is regarded as a clinically significant improvement. Therefore, the sample size in this trial can be estimated initially according to PASS software. Each group needs at least 172 patients (assuming a 15% dropout rate), 1200 patients in six groups will be enrolled in the study.

Baseline assessments before the start of treatment include neurological examination, grading of facial function, measurement of ipsilateral pain, documentation of concurrent medication. Patients with acute, unilateral, peripheral facial palsy, who are referred from neurology, internal medicine or emergency departments, or who seek care directly, are screened by physicians in 16 public county hospitals and Xijing hospital in Shaanxi province. There is at least one experienced neurology physician, who is responsible for implementing the study, with a special interest in facial palsy in each hospital. A written consent form will be obtained from each patient and retained by the investigators.

#### Inclusion criteria

Patients with Bell' s palsy meeting the following inclusion criteria are enrolled in the study: involvement of unilateral facial nerve paralysis only, aged between 18 and 75 years old, period of onset of facial paralysis within 72 h.

#### Exclusion criteria

Patients with any of the following are excluded: pregnancy, breastfeeding, being a woman of child bearing age who is unwilling to use contraceptives during the medication period, other neurological diseases, diabetes, badly controlled hypertension, current or history of serious heart disease, history of renal or hepatic disease, gastric or duodenal ulcer, history of glaucoma, acute otitis or history of ipsilateral chronic otitis, history of tuberculosis, history of immunodeficiency syndromes, recent head injury, psychiatric disease, or any other condition that is at risk of being influenced by the study medication or that might have affected completion of the study.

### Randomization

Patients are to be randomly assigned to one of six treatment groups. A computer-generated allocation sequence (in permuted blocks with a block size of 12) will be created by an operator who is not directly involved with the study. Once created, the allocation sequence will be kept in a secure locked drawer making it inaccessible to all study personnel. Allocation concealment will be achieved using sequentially numbered sealed envelopes which are opaque when held to the light. When a patient is enrolled in the study, an independent researcher will be contacted and asked to give the next numbered envelope to the physician who will make up the medications to be used in the study. The independent researcher will record the patient's details and the number of the envelope assigned to that patient. This procedure ensures that the randomization is not influenced by the researchers taking part in this study.

### Interventions

Patients will be randomized to one of six treatment arms: placebo prednisolone, prednisolone, placebo prednisolone plus acupuncture (acute stage), prednisolone plus acupuncture (acute stage), placebo prednisolone plus acupuncture (resting stage), or prednisolone plus acupuncture (resting stage) within 72 h after onset of Bell's palsy.

Drug treatment: Prednisolone as 5 mg tablets will be given within 72 h after onset of Bell's palsy as a single dose of 40 mg daily for 5 days; the dose will then be reduced by 10 mg per 5 day, with a total treatment time of 20 days.

Acupuncture therapy: On the basis of previous studies, the acupuncture points used will be Dicang (ST4), Jiache (ST6), Yangbai (GB14), Xiaguan (ST7), Taiyang (EX-HN5), Quanliao (SI18) and Yifeng (TE17) on the affected side, and Hegu (LI4) bilaterally. A qualified acupuncturist will give the acupuncture in every center. For acute stage acupuncture, shallow puncturing will be used at facial acupoints and routine puncturing will be used at other acupoints within 72 h after onset of Bell's palsy. Yifeng (TE17), Hegu (LI4) will be punctured 0.5-1.0 cun, the others will be punctured 0.1-0.3 cun. For resting stage acupuncture, penetrative needling will be used from Dicang (ST4) to Jiache (ST6) and from Taiyang (EX-HN5) to Quanliao (SI18) 2-3 cun, and routine puncturing will be used at other acupoints 7 d after enrolment. Filiform needles (33 - 49.5 mm, 0.32 mm) will be used with moderate stimulation to get an acupuncture sensation, and the needles will be retained for 30 minutes, once a day, five times a week, for a total period of four weeks. The locations of those points are defined in 1993 by the World Health Organization (WHO), Regional Office for the Western Pacific [15].

### Outcomes

In order to reduce outcome assessment bias, the investigators should be given specific training for facial grading, and both the Sunnybrook and House-Brackmann scales will be used.

#### Primary outcome

The primary outcome measurement in this study is the recovery of facial function before randomization and 7 days, 17 days, 1 month, 2 months, 3 months, and 6 months after randomization. Facial function will be assessed at all visits with two grading systems (The Sunnybrook system and The House-Brackmann scale). If recovery is complete (Sunnybrook scale score of 100 points) at 2 or 3 months, the next follow-up grading will be at 6 months.

#### Secondary Outcome

Pain around the ear, in the face, or in the neck will be registered on a visual analogue scale that ranges from 0 to 10 points, where 0 is no pain and 10 very severe pain. Occurrence of facial spasm or contracture and synkinesia in the different treatment arms at any time will be observed and recorded.

### Statistics

The study will be performed using intention to treat. In this analysis, all randomized patients will receive at least one therapy, but patients who did not start treatment will be excluded (modified intention-to-treat analysis). The last-observation-carried-forward method will be used for the modified intention-to-treat analysis. The missing data points will be imputed in the post-baseline follow-up visits from the last observation available for each patient. An interaction test was done to show the synergistic effect of the combination of prednisolone and acupuncture. Categorical variables will be compared by using Fisher's exact test. Cox proportional hazards models will be used to estimate the hazard ratio (HR) of recovery, including 95% CI. These will be compared between groups by using t-test, and Mann-Whitney, or Chi-squared tests as appropriate. All statistical assessments are two-sided, and a P value < 0.05 is considered statistically significant.

## Discussion

Bell's palsy is a common disease. Recent evidence from large randomized controlled trials indicates that corticosteroids are associated with a reduced risk of un-satisfactory recovery in Bell's palsy. Acupuncture, a therapy of Chinese traditional medicine, has been recognized as an alternative treatment method for many diseases. Its effectiveness for Bell's palsy has been shown by a few clinical trials in recent years [7-10]. However, the best intervention time for acupuncture has been uncertain to date. Many studies testified the effectiveness of staging acupuncture by comparing results with non-staging acupuncture [14-18]. Although some research results showed that there were differences between staging acupuncture and non-staging acupuncture to treat Bell's palsy, these trials had some methodological defects. For instance, four clinical controlled trials illustrated that the therapeutic effect of acupuncture treatment at different path-stages was better than that of routine acupuncture treatment for treating facial paralysis [16-19]. Randomization method was not used in these trials, and the sample size was smaller than 80 clinical cases in two studies [16,17], the biggest one being less than 130 clinical cases [18,19]. In addition, the side effects during acupuncture treatment and the follow-up tests were not reported in any of these trials. The design of the control group was illogical as well [20]. So which method is the best treatment at different path-stages for Bell's palsy needs to be investigated. Staging acupuncture, when administered with corticosteroids, may also be associated with additional benefits. In summary, the purpose of this trial is to provide evidence of the effectiveness of staging acupuncture to treat Bell's palsy, and to find out the best approach for treating this disease.

We expect to complete recruitment by December 2011 and anticipate that the results from this study will have implications for health care resourcing and will facilitate improvements in the treatment of people with Bell's palsy. The results of this study will also allow us to determine, for the very first time, if a prednisolone intervention alone is as effective at facilitating facial nerve recovery as an intervention involving both prednisolone and staging acupuncture. These results may have important implications for the design of sustainable cost-effective health services for people with Bell's palsy.

Central randomization is used in our trial, which is a strict, complete randomization, and it ensures adequate concealment. During the whole trial, the patients are not aware of which group they are to be assigned to, but they do know they will receive acupuncture treatment at different path-stages. Additionally, this trial is a multicenter randomized controlled study, with six months' follow up, containing a large number of patients. Up to now, our trial is one of the largest randomized controlled trials addressing the effectiveness of prednisolone in combination with staging acupuncture treatment for Bell's palsy.

## Competing interests

The authors declare that they have no competing interests.

## Authors' contributions

GZ conceived the study; FX designed the study protocol and wrote the manuscript; GZ, LH and SW sought funding and ethical approval; XL, KW and ZJ are responsible for the statistical analyses. All authors have read and approved the final version of the manuscript. They are the coordinators of the clinical centers that will enroll the trial participants. The corresponding author had final responsibility for the decision to submit for publication.
