# Erratum to: Prednisolone and acupuncture in Bell’s palsy: study protocol for a randomized, controlled trial

**DOI:** 10.1186/1745-6215-15-315

**Published:** 2014-08-08

**Authors:** Feng Xia, Junliang Han, Xuedong Liu, Jingcun Wang, Zhao Jiang, Kangjun Wang, Songdi Wu, Gang Zhao

**Affiliations:** Department of Neurology, Xijing Hospital, The Fourth Military Medical University, Xi’an, Shaanxi China; Department of Neurology, Central Hospital, Hanzhong, Shaanxi China; Department of Neurology, The First Hospital, Xi’an, Shaanxi China

## Correction

Following publication of this protocol [1], it was brought to the Editorsâ€™ attention in May 2014 that design of the study has been changed from a randomized controlled trial to a non-randomized study, and so will no longer follow the published protocol. For more information on the updated design and conduct of the study, please contact the corresponding author.
